# Health in All Policies at the Local Level: What Facilitates Success? Comment on "A Realist Explanatory Case Study Investigating How Common Goals, Leadership, and Committed Staff Facilitate Health in All Policies Implementation in the Municipality of Kuopio, Finland"

**DOI:** 10.34172/ijhpm.2023.7975

**Published:** 2023-08-21

**Authors:** Carmel Williams, Nicole Valentine

**Affiliations:** ^1^Centre of the Health in All Policies Research Translation, School of Public Health, University of Adelaide, Adelaide, SA, Australia; ^2^Health Translation SA, South Australian Health and Medical Research Institute (SAHMRI), Adelaide, SA, Australia; ^3^WHO Collaborating Centre on Advancing Health in All Policies Implementation, Adelaide, SA, Australia; ^4^Social Determinants of Health, World Health Organization (WHO), Geneva, Switzerland

**Keywords:** Health in All Policies, HiAP, Social Determinants of Health, Public Policy

## Abstract

The paper by Guglielmin and colleagues^1^ examines the implementation of Health in All Policies (HiAP) in a local government context in Kuopio Finland. The authors use a realist explanatory case study design to explore what has supported HiAP implementation with a focus on two specific hypotheses on what leads to success: common goals and committed leadership and staff. The paper is well argued using appropriate methodology and their findings support the importance of the success factors tested by their two hypotheses. However, the narrowed focus on just two hypotheses underrepresents the complexity of implementing HiAP at any level of government, including local government. Given its local government focus, the paper would have been strengthened by referencing the lessons gained from the Healthy Cities movement. Local government is a critical setting for action to address health and health equity and there is great potential to continue research that adds to the knowledge base on how to successful implement HiAP. Finally, it is important to acknowledge that Finland has a unique HiAP history. It is recognised as a global leader in the field, and the role of local government in Finland differs from many other countries. These factors may impact on the transferability of the case study findings.

 Globally, there has been increasing movement within countries to apply approaches that increase healthy public policy, with the aim of delivering improved population health outcomes. Healthy public policy has long been recognised as an important lever for health systems, to use to influence the policy decisions made by governments, especially with agencies outside of heath. Public policy decisions of government have a significant impact on population health and wellbeing, as these decisions shape the distribution of the causes of the causes of disease, the underlying determinants of health and health equity.^2^ Health sector actors need to engage with the policy decision-making process of government if they are to inform, influence and ultimately shape the policy decisions of other government agencies.^3^ Working across government ministries, which tend to operate in vertical silos, is challenging. Health in All Policies (HiAP) is an approach that aims to support the health system to systematically engage in the policy making process of government.

 HiAP aims to support traditional areas of government programs and services such as education, urban planning and environmental health and safety. Importantly, HiAP aims to work beyond these areas to apply a health lens to public policy design on the issues that affect living, learning and work settings, such as the structure of the labour market, and how society deals with distributive mechanisms affecting access to basic resources needed for health and health equity. These policy areas have only been considered as “health” issues by a minority. The increasing evidence on the interconnections between human activity, population health, inequalities and the health of the planet (alongside the rise of well-being economic thinking^4^), is changing these perceptions, where the concept of healthy public policies is becoming more mainstream.

 There are advantages of working on HiAP at the local level.^5^ For example, in comparison to national and sub national governments, authorities working within local government operate in closer proximity to the community and are therefore better positioned to engage with their needs and respond to challenges and opportunities more actively.

 Governments systems tend to be constructed, in hierarchical vertical silos which is a key challenge for achieving healthy public policy outcomes and this is true regardless of the level of government national, sub-national or local. HiAP approaches lay out a framework of strategies, actions and processes designed to break through the siloed structures, both vertically and horizontally, and provide a bridge to the policy making process.

 The research to understand how HiAP approaches operate and what constitutes success is building. Much of the research has focussed at the state or national level, as Guglielmin and colleagues point out. Baum and colleagues undertook a 5-year evaluation of the South Australian HiAP approach and found a range critical factors that supported its implementation.^6^ In 2018, Shankardass and colleagues developed a framework for evaluating the implementation of HiAP that aims to take account to the complex political environment in which HiAP approaches operate called *HARMONICS*. Guglielmin with Shankardass and colleagues have adapted this framework in the Kuopio Case Study.^7^

 A key observation put forward by the paper’s authors is that local government is an under-researched area in the HiAP field.^5^ While agreeing with this general point, it is also important to observe that documentation and research of HiAP at all levels, including the local level, is a growing area.^5^ There are several reports documenting cases studies and research on HiAP operating at the national and subnational levels of government. For example, the Global Status Report produced in 2019 by the Global Network for Health in All Policies documented 41 examples of HiAP practice at national, sub national and the local level, with 6 examples originating from the local level.^8^ In the United States, the National Association of County and City Health Officials developed a report in 2017 on 14 local governments including from cities.^9^

 In the academic literature there have been studies in Scandinavia and the Netherlands, examining HiAP of the local level, and these provide useful insights several of which are confirmed by the study by Guglielmin and colleagues.^1^

 Literature on HiAP is also increasing in the context of the World Health Organization (WHO) Healthy Cities movement, which has a long-standing history of working through the local level of government to improve health, wellbeing and equity. It is slightly surprising that the authors did not refer to these examples.

 In moving beyond descriptive studies to explanatory ones, to understand the mechanisms at play, mixed method evaluations are clearly needed, and the methodological efforts of Guglielmin et al^1^ are exemplary in this respect. The use of realist case study methods appears to be an appropriate way to research HiAP. HiAP initiatives operate in a highly political environment and are therefore difficult to research using traditional research methods.^10^ Case study methods offer the opportunity to unpack some of the activities in a detailed and sensitive manner. However, the research methods could have been strengthened by expanding the number of hypotheses and success factors under investigation. The HiAP approach supports actors and actor-groupings, who do not behave in predictable linear patterns, to work across organizational hierarchies, cultures, and disciplines to generate improved solutions to complex problems. As such it is itself complex intervention. Analytical frameworks using complexity concepts like structural and relational components affected by dynamic feedback loops can also enhance the framing of hypotheses.^11^

 The narrowing of the hypothesis to just two key hypotheses namely, (1) The existing of common goals between agencies and (2) that leadership and staff are committed to HiAP approach, limited the breadth of the case study. The authors decision to focus on just two key factors, despite evidence from the literature identifying multiple factors involved in successful HiAP implementation, is unclear. While the two hypothesises were informed from a scoping review of HiAP at the local government, the authors own argument that local government is under researched suggests that there will be limited evidence available. However, if they had expanded the scoping review to include all HiAP initiatives operating at the national, sub national level as well as the local level and from the healthy cities field, they will have identified a wider range of success factors that are considered instrumental in HiAP implementation. The reduction of success factors to just two key areas, narrows the results and limits the value of the case study paper.

 Through these two hypotheses, the authors in fact identify three important success factors – “strong supporting evidence for the hypotheses that having common goals between sectors, and that local leadership and committed staff, facilitate intersectoral work for health.” These three factors are also mentioned in the new WHO HiAP model, along with other important factors. While these success factors align with other research on the implementation of HiAP, they do not cover all the conditions required for successful HiAP implementation. For example, the accountability and governance processes and structures that are needed to enable cross sector collaboration, the finances and budget and skills required to work collaboratively. The authors point out the importance of the context. Part of that context is the importance of the culture within organisations and governments that make collaborating possible.^12^

 These additional HiAP success factors are documented in the new HiAP 4 Pillars Model being put forward in a publication of the WHO for testing and refinement by practices in countries.

 In summary, the new HiAP model:

- outlines the organizational structures and mechanisms required to build collaboration;
- acknowledges the social determinants of health framing and the structural drivers of health inequity to frame the scope of policies and inclusion of the equity goal;
- applies to any public policy and/or health issue that requires multisectoral collaboration;
- is adaptable and relevant to different countries and political contexts;
- promotes the sustainability of a HiAP approach and its focus on public value; and
- connects HiAP to achievement of the Sustainable Development Goals, sustainability, and social development with equity.

The New HiAP Model has at the centre, the “four pillars” and these focus on the functions and capacities needed to apply a collaborative HiAP approach.^13^ Many of these functions are relevant to sustaining multisectoral collaboration regardless of the issue of focus or the level of government and they include supporting common goals, committed leadership and staff, proven success features in the Kuopio study.

 HiAP operates within the political and policy decision-making environment of governments and in these circumstances context matters. It was important that the authors acknowledged the special context of Finland, especially its political and social history of egalitarianism and social democracy. In addition, the authors identify the socio-economic circumstances of Kuopio as a high social economis status municipal area, with a history of collaboration and intersectoral action. Finland is also recognised as a global leader in the field of HiAP and the commitment from the Finnish government to health, wellbeing and equity is longstanding and nonpartisan. This unique history provides important background that may make implementing HiAP approaches smoother in the Finnish context. The autonomy and responsibility given to local governments in the Finnish context may be less transferable to many other countries, and so it would have been helpful if it was also emphasised.

 Finally, it would have been interesting if the case study methods could have been extended to a second and /or third Finnish local government area, perhaps including one that has a large low social economis status population. If the findings were consistent across these different local government areas, the generalisability of the findings would be strengthened, which may increase the potential for the findings to be transferable globally. Furthermore, the inclusion of equity-related values that enable explicit comparison between Kuopio and another municipality, would be interesting. In other Scandinavian countries, the difficulty of evidence on health and determinants inequalities at the local level has been raised as a barrier to action.^14^

 The paper “A realist Explanatory Case Study Investigating How Common Goals, Leadership and committed Staff, Facilitate Health in All Policies in the Municipality of Kuopio Finland” is a useful addition to the evidence base on how to successfully implement healthy public policy approaches such as HiAP within a local government context. Increasing HiAP action at the local government level offers strategic opportunities to influence the health and wellbeing of local communities across the globe, through informing, influencing and shaping the public policy decisions of local government decision-makers.

## Ethical issues

 Not applicable.

## Competing interests

 Authors declare that they have no competing interests.
